# Genome-wide DNA methylation signature predict clinical benefit of bevacizumab in non-small cell lung cancer

**DOI:** 10.1186/s12885-022-09918-1

**Published:** 2022-07-29

**Authors:** Butuo Li, Chao Jiang, Yiyue Xu, Xinyu Fan, Linlin Yang, Bing Zou, Bingjie Fan, Linlin Wang

**Affiliations:** 1 Department of Radiation Oncology, Shandong Cancer Hospital and Institute, Shandong First Medical University and Shandong Academy of Medical Sciences, 440 Jiyan Road, Jinan, 250117 Shandong China; 2 Department of Otorhinolaryngology Head and Neck Surgery, Shandong Provincial Hospital Affiliated to Shandong First Medical University, Jinan, Shandong China

**Keywords:** Bevacizumab, Non-small cell lung cancer, Methylation, Signature, Predictive

## Abstract

**Background:**

The efficacy of bevacizumab in non-small cell lung cancer (NSCLC) patients is unsatisfactory, and the selection of suitable patients is still challenging. Given the epigenetic modifications can contribute to an aberrant regulation of angiogenesis and microenvironment, we investigated DNA methylation profiles to determine clinical benefit of bevacizumab in NSCLC patients.

**Methods:**

Genome-wide DNA methylation profiling was performed in NSCLC patients treated with chemotherapy in combination with bevacizumab. Patients were divided into better prognosis group (A group) and inferior prognosis group (B group) based on their survival. The difference of methylation patterns and respective functional enrichment analysis were performed between two groups. Prognostic DNA methylation signature for bevacizumab was established with the least absolute shrinkage and selection operator regression analyses. TISIDB database was further used to infer immunological relationship for prognostic related DNA methylation.

**Results:**

Twenty patients were included in this study, and significantly distinct methylation patterns were observed between patients with different prognosis. Related genes of different methylation regions were significantly enriched in the biological process of cell projection assembly, neutrophil mediated immunity, and pathway of VEGFA-VEGFR2 signaling pathway, neutrophil degranulation. A 10-gene DNA methylation signature for prognosis prediction was established with the C-index of 0.76. And host genes of signature were found to be related to the abundance of ActCD4, Th1, ActCD8, NKT and neutrophil cells.

**Conclusion:**

The 10-gene DNA methylation signature could serve as a novel biomarker to predict the clinical benefit of bevacizumab therapy and improve this anti-tumor approach for NSCLC patients.

**Supplementary Information:**

The online version contains supplementary material available at 10.1186/s12885-022-09918-1.

## Introduction

Tumor neovascularization which is primarily composed of endothelium-dependent angiogenesis and vasculogenesis, is essential for tumorigenesis, progression, and metastasis [1]. Anti-angiogenesis treatment, such as bevacizumab and anlotinib, was identified as a promising therapeutic approach by regulating the balance of pro-angiogenic and anti-angiogenic factors. And the combinational efficacy of anti-angiogenic and chemotherapy was regarded to be attributed to vascular normalization followed by the transmission of chemical agents to the tumor [2].

Bevacizumab, a humanized monoclonal antibody that targets VEGF, in combination with chemotherapy ± immunotherapy is a treatment option for advanced non-squamous non-small cell lung cancer (NSCLC) patients [3, 4]. The clinical benefit with bevacizumab has been observed in several phase III clinical trials, however the survival of these patients was still far from satisfactory, with the median overall survival (OS) ranging from 12.3 months to 24.3 months when combined with chemotherapy [3, 4]. Thus, the biomarkers are urgently needed to clearly distinguish patients who have a chance of benefiting from bevacizumab.

Accumulated evidence has indicated that the primary or acquired resistance to anti-angiogenesis which undermined the clinical application of anti-angiogenic strategies. Unfortunately, promising biomarkers like VEGF-A, VCAM-1, bFGF [5, 6], or clinical markers such as treatment-related hypertension [7] failed to demonstrate its utility and reproducibility. The potential explanations for the unsatisfactory effect of anti-angiogenetic therapy include the high adaptability of tumor microenvironment. Besides, NSCLC is known as a highly vascularized tumor, and cancer cells start to grow along with the existing vessels to obtain sufficient essential nutrients and gases without the need to form new vasculature. Given the multiple mechanism of anti-angiogenesis and complexity of microenvironment of tumor, no single marker could achieve precise prediction of efficacy.

DNA methylation, known as an important epigenetic modification, annotates genomic regions which plays important role in gene transcription and expression. The pattern of DNA methylation alterations which are locus dependent, are considered to be involvement in lung cancer carcinogenesis [8] and development of drug resistance [9]. Promoter methylations, a common event in NSCLC, are fit for tracking the signals due to their early and persistent existence in cancer development [10].

Previous studies have demonstrated DNA methylation-based biomarkers for prognosis prediction and response to conventional therapy in NSCLC patients [11, 12], and the certain influence of methylation pattern on the response to antiangiogenic therapy in breast cancer patients [9]. To our knowledge, there is currently no related research exploring the association between DNA methylation and the prognosis in NSCLC cancer patients. Thus, we set out to determine whether a profile of DNA methylation can predict the clinical response to bevacizumab in patients with NSCLC.

## Result

### Clinical characteristics of patients

Twenty patients receiving bevacizumab and chemotherapy were enrolled in this study, and ten patients in each group. To define the epigenomic characteristics associated with patients who would gain clinical benefit from bevacizumab treatment, we collected tissue samples from these patients before bevacizumab treatment. Those patients assigned to A group showed prolonged progression-free survival (PFS) (19.3 vs 5.0 months, *P* < 0.001) and OS (Not available vs 9.8 months, *P* = 0.02) compared to patients in B group. The median age of all patients was 57, and the demographics and clinical characteristics were shown in Table 1.

**Table 1:** Demographics and disease characteristics of patients

Total 20 patients	Inferior prognosis group (B group)	Better prognosis group (A group)	P
Age			0.178
≤57	4 (40%)	7 (70%)	
> 57	6 (60%)	3 (30%)	
Gender			0.025
Female	3 (30%)	8 (80%)	
Male	7 (70%)	2 (20%)	
Smoking History			0.051
No	5 (50%)	9 (90%)	
Yes	5 (50%)	1 (10%)	
Anatomical type			1
Central	3 (30%)	3 (30%)	
Peripheral	7 (70%)	7 (70%)	
EGFR status			0.079
Sensitive mutation	2 (20%)	5 (50%)	
Negative	3 (30%)	5 (50%)	
Resistance mutation	2 (20%)	0 (0%)	
NA	3 (30%)	0 (0%)	
Bone metastasis			0.606
No	8 (80%)	7 (70%)	
Yes	2 (20%)	3 (30%)	
Brain metastasis			0.136
No	10 (100%)	8 (80%)	
Yes	0 (0%)	2 (20%)	

### Differential DNA methylation pattern between patients with good prognosis and poor prognosis

Firstly, the MeDIP-seq libraries were constructed with the DNA derived from sample of NSCLC patients receiving bevacizumab. After data pre-processing, all libraries exhibited the main peak of ~ 298 bp containing the ~ 120 bp sequencing adapters as expected. Illumina Hiseq 4000 was used to perform the sequencing of DNA MeDIP-seq libraries. The minimum of 10 million and 18.5 million unique mapped reads, which were mapped to the reference genome (Human hg38), were achieved from patients in A and B group, respectively.

**Fig. 1:**
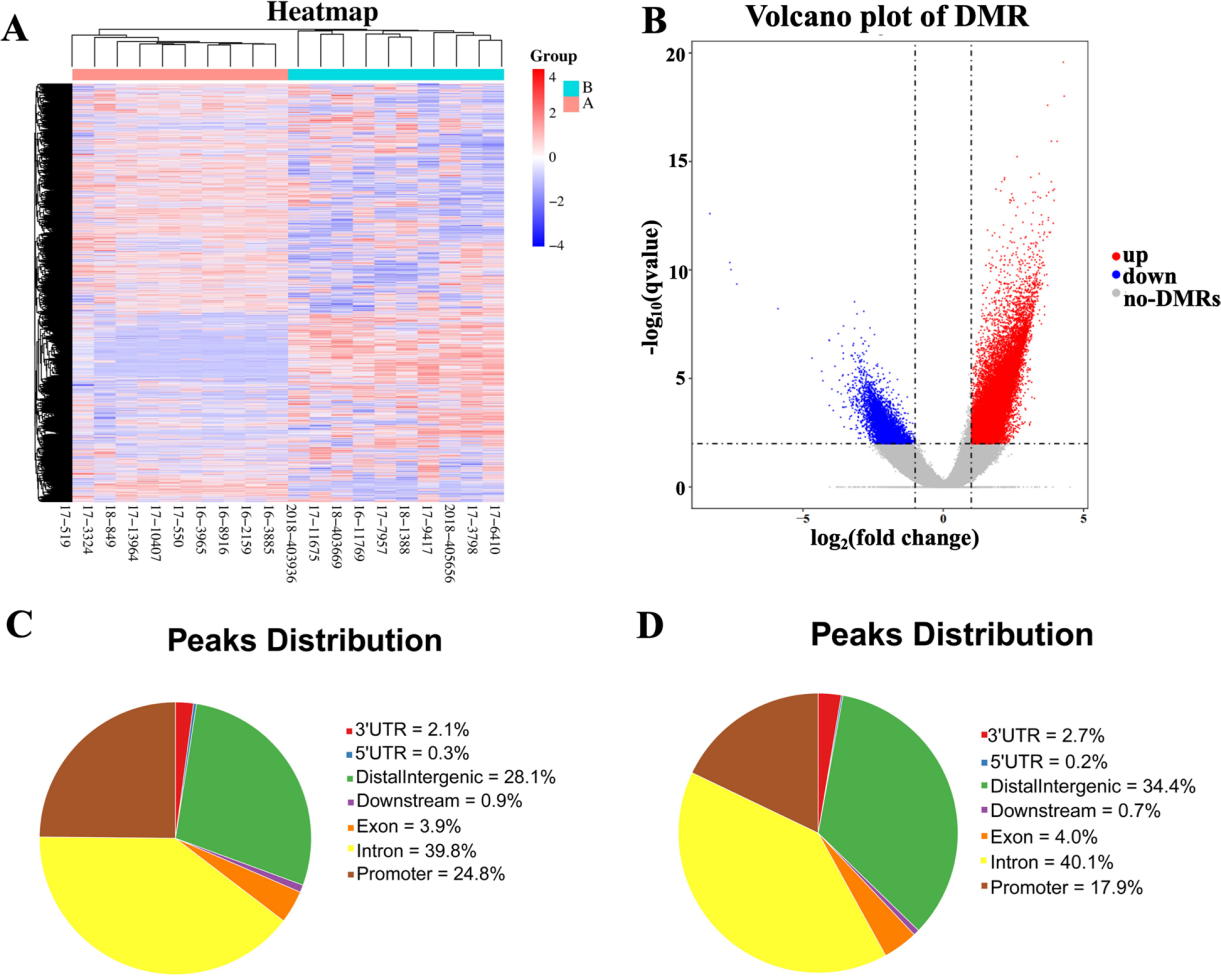
The DNA methylation patterns between patients with good prognosis and poor prognosis. **A**. Heatmap of 9517 DMRs located on promoter regions between A group and B group. The color of the heatmap means the level of methylation of DMRs, the red color means the hypermethylation and blue color means the hypomethylation. **B**. The volcano plot of hypermethylated region and hypomethylated region. **C**. The distribution of hypermethylated region located in exon, intron, promoter, distal intergenic and other genomic features. **D**. The distribution of hypomethylated region located in exon, intron, promoter, distal intergenic and other genomic features

As shown in Fig. 1A, significantly distinct methylation patterns were observed between A and B group patients based on the clustering analysis results. DESeq2 method (FDR < 0.01 and |log2(fold change)| > 1) revealed 40,412 DMRs between two groups, 33,183 of which were hypermethylated and others were hypomethylated (Fig. 1B). The distribution of differentially methylated sites in the whole genome including exon, intron, promoter, distal intergenic and so on, were displayed in Fig. 1C and D. DNA promoter methylation, which was defined as 3Kb upstream of transcription start site and might induce the altered gene expression [13], was the third most common methylated region. Distal intergenic, which represented the intergenic region other than the promoter and downstream of genes, was the second most common methylated region. And intron of gene was the most common methylated region.

### Functional enrichment analysis of differential promoter methylation

DMRs on promoter regions were further analyzed in consideration of the significant role of promoter methylation in cancer development and progression. Total of 9517 DMRs were found to locate on promoter regions, corresponding to 6419 genes. Univariate cox regression analysis among 6419 genes indicated that there were 1464 genes related to the survival of NSCLC patients receiving bevacizumab.

**Fig. 2:**
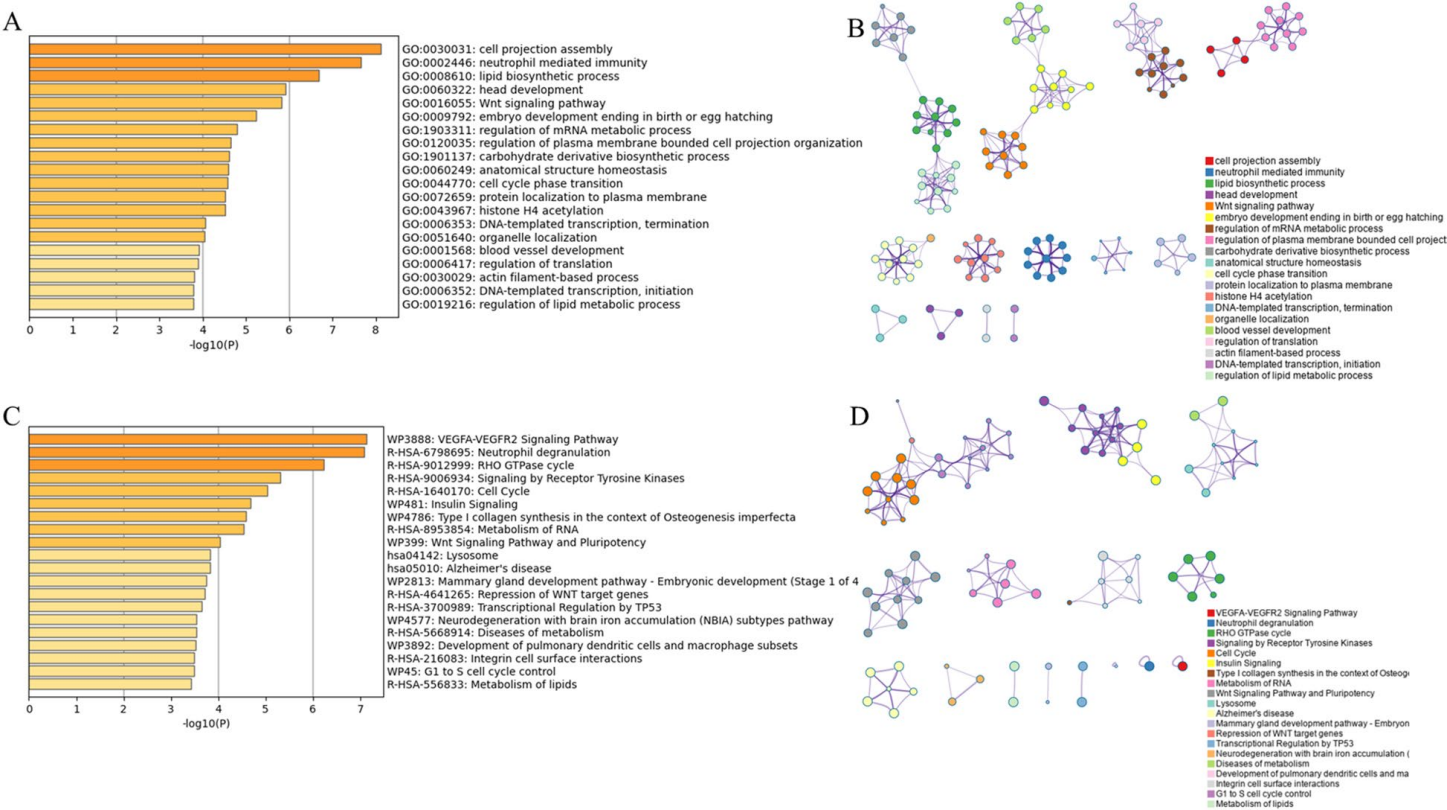
Functional enrichment analysis of survival-related host genes of promoter-associated DMRs. **A**. The top 20 enriched terms in GO analysis colored by *P* value. **B**. Network of GO enriched terms colored by cluster ID. Circles with different colors indicated membership in different GO term, and lines between them indicated the connection between different terms. **C**. The top 20 enriched pathways colored by *P* value. **D**. Network of pathways colored by cluster ID. Circles with different colors indicated membership in different pathways, and lines between them indicated the connection between different terms pathways

In order to understand the overall functional relevance of these genes, GO analysis and pathway analysis were further performed. Regarding to the biological processes (BP), related genes of DMRs on promoter regions were significantly enriched in the cell projection assembly, neutrophil mediated immunity, lipid biosynthetic process, head development and Wnt signaling pathway as shown in Fig. 2A and B. And pathway analysis revealed that related genes enrichment mainly occurred in VEGFA-VEGFR2 Signaling Pathway, neutrophil degranulation, RHO GTPase cycle, Signaling by Receptor Tyrosine Kinase and Cell Cycle as shown in Fig. 2C and D. It’s worth noting that potential relationship between the efficacy of bevacizumab and neutrophil related immunity and biology, apart from VEGFA-VEGFR2 Signaling.

### Establishing a genome-wide DNA methylation signature for prognosis prediction

**Fig. 3:**
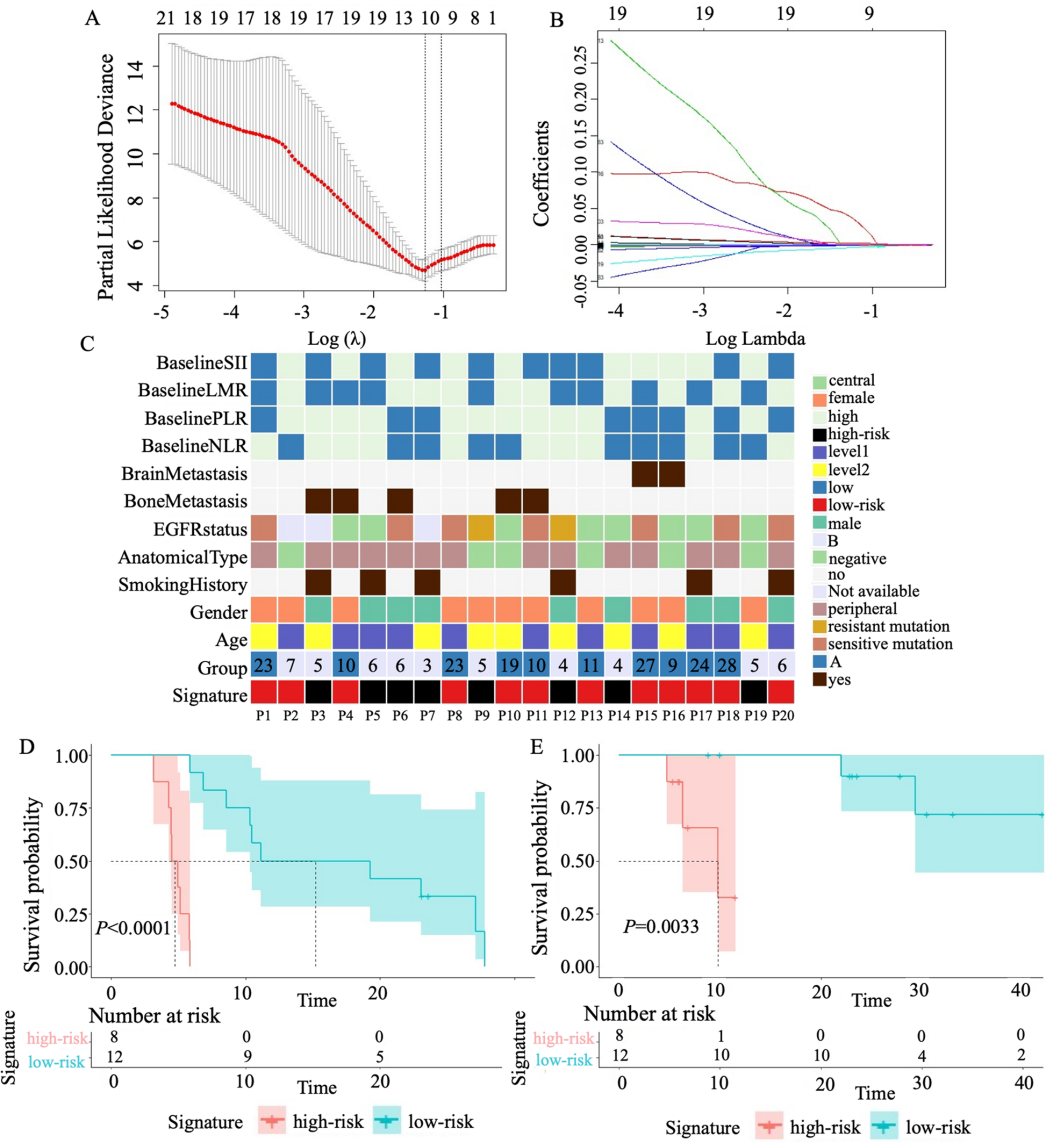
Construction and analysis of genome-wide DNA methylation signature. **A**. Partial likelihood deviance of host genes revealed by the LASSO-Cox regression model. The red dots represented the partial likelihood of deviance values, the gray lines represented the standard error (SE), the two vertical dotted lines on the left and right represented optimal values by minimum criteria and 1-SE criteria, respectively. 1-SE criteria was used to select host genes in model. **B**. LASSO coefficient profiles of the survival-related host genes. **C**. The clinicopathological characteristics of patients in different risk signature subgroups. The number in the row of group indicated the PFS of patient. **D**. Kaplan-Meier analysis of patients with high-risk signature and low-risk signature for PFS. **E**. Kaplan-Meier analysis of patients with high-risk signature and low-risk signature for OS

A total of 1464 genes from univariate cox analysis among 6419 genes were included in least absolute shrinkage and selection operator (LASSO) -Cox analysis in order to establish methylation signature associated with the efficacy of bevacizumab (Fig. 3A and B). Of these, ten related genes of DMRs on promoter regions were selected as the optimal genes for the predictive signature, including *TMEM222, VTCN1, R3HCC1L, VPS51, POLR3B, CCDC154, HOXB2, MSI2, TTC21A, PTH1R*, whose coefficient was 0.0020294600, − 0.0004274544, − 0.0001309280, 0.0152809600, − 0.0004984510, − 0.0002155878, − 0.0000818177, − 0.0024625390, − 0.0004356569, − 0.0000590949, respectively.

The zero score of methylation signature was selected as cutoff value to define the high-risk and low-risk group, which provided a novel classification to identify patients who might benefit from bevacizumab. Patients with level of methylation signature higher than zero were classified as high-risk and others were classified as low-risk. Finally, 12 patients were assigned to low-risk group and 8 patients were assigned to high-risk group. As shown in Fig. 3C, patients in low-risk group were highly consistent to A group patients (*P* < 0.001). Besides, male and elder patients were more likely to be assigned to high-risk group.

Survival analysis indicated that the high level of DNA methylation signature was significantly associated with the inferior PFS (median PFS 11.1 vs 4.5 months, *P* < 0.001) and OS (median OS NA vs 9.8 months, *P* = 0.003) of NSCLC patients receiving bevacizumab (Fig. 3D and E). The C-index of the DNA methylation signature was 0.76, implying the high predictive accuracy of DNA methylation signature. Multivariate cox analysis also indicated the independent significant predictive effect of this DNA methylation signature (Table 2).

**Table 2:** Univariate and multivariate cox analysis for PFS

Variables	Uni HR	95% CI	*p*-value	Multi HR	95% CI	*p*-value
Age						
≤ 57	1					
>57	2.4	0.9-6.6	0.081			
Gender						
Female	1					
Male	1.5	0.55-3.9	0.45			
Smoking History						
No	1					
Yes	2.0	0.7-6	0.19			
EGFR status			0.026			
Sensitive	1					
Negative	2.27	0.6-7.96	0.20			
Resistance	2.98	2.3-171.1	0.007			
Anatomical type						
Central	1					
Peripheral	0.64	0.23-1.8	0.41			
Bone metastasis						
No						
Yes	1.5	0.5-4.3	0.49			
Brain metastasis						
No	1					
Yes	0.67	0.15-3.0	0.6			
DNA methylation signature						
High-risk	1			1		
Low-risk	0.023	0.0026-0.21	< 0.01	0.036	0.003-0.372	0.005

### Immune cell abundance associated with the DNA methylation signature

**Fig. 4:**
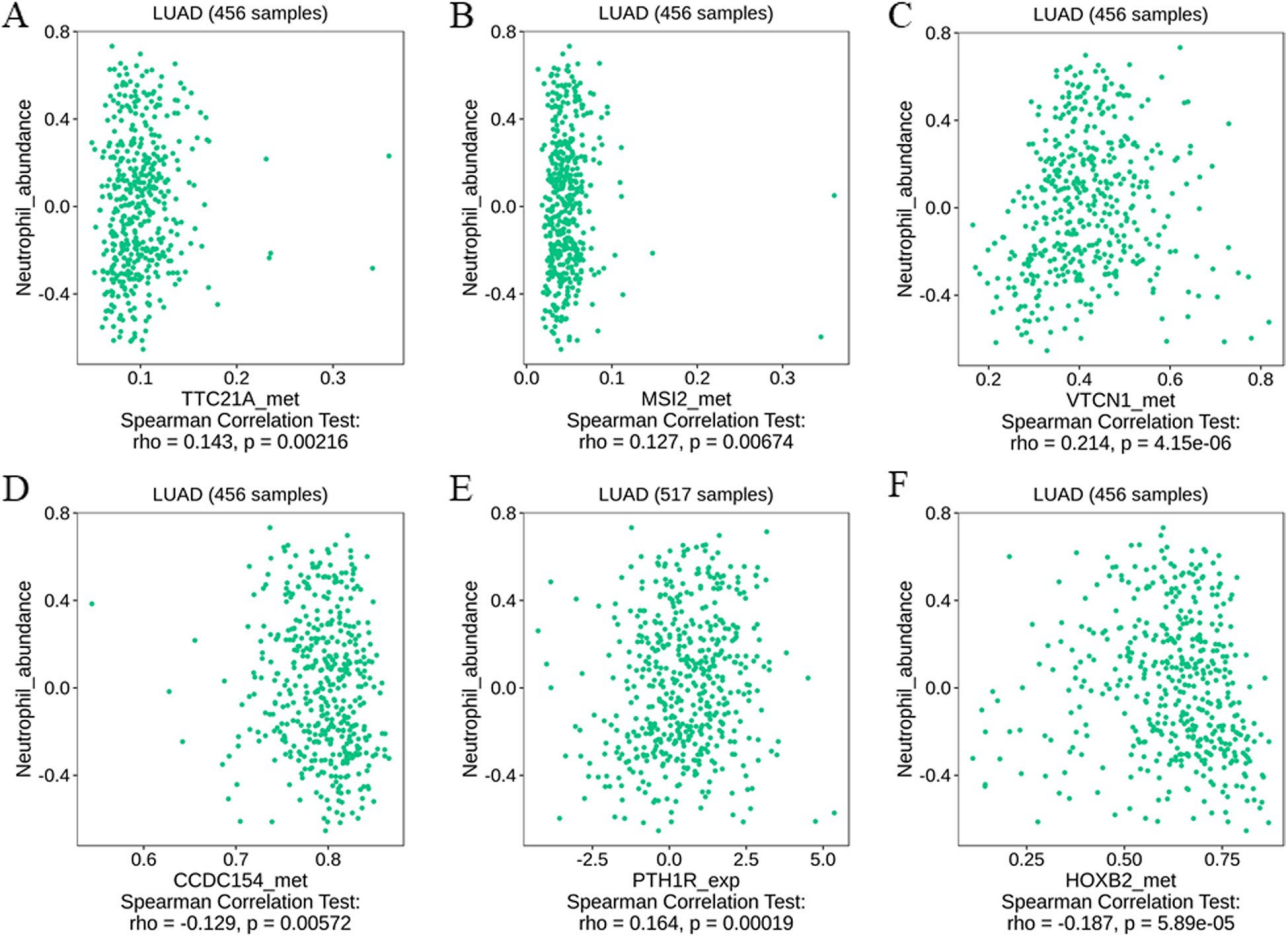
The association between abundance of neutrophil cell and host genes in DNA methylation signature. **A**. *TTC21A*. **B**. *MSI2*. **C**. *VTCN1*. **D**. *CCDC154*. **E**. *PTH1R*. **F**. *HOXB2*

On account of important role of tumor microenvironment for the effect of anti-angiogenesis, we further analyzed the immune cell abundance associated with the DNA methylation signature in LUAD patients of TISIDB database. Abundance of ActCD4, Th1, ActCD8 cells were found to be related to methylation of *TTC21A, MSI2, CCDC154, VTCN1, POLR3B, PTH1R, HOXB2*. And abundance of NKT and neutrophil cell (Fig. 4) were found to be related to methylation of *TTC21A, MSI2, CCDC154, VTCN1, PTH1R, HOXB2.*

## Discussion

The widespread use of anti-angiogenesis, such as bevacizumab and anlotinib, has markedly improved the survival of patients with advanced NSCLC. However, a substantial percentage of patients do not get clinical benefit from bevacizumab [3, 4]. The results from IMpower 150 also indicated that only 55% of NSCLC patients could receive clinical benefit even combined with immunotherapy [14]. This means that patients might experience adverse effect from anti-angiogenesis without benefit. Thus, it is essential to explore the biomarkers to predict the outcome of anti-angiogenesis. However, no biomarker has been identified that would enable the personalized use of bevacizumab. The potential biomarker for bevacizumab efficacy has been investigated in a range of indications include plasma VEGF, VEGF-A, angiopoietin 2, hepatocyte growth factor, placental growth factor, microvascular density, interleukin 6 and 9, however no consistent or conclusive results were obtained [15]. Radiomics features were also investigated as non-invasive markers for prognosis prediction of bevacizumab [16]. Despite intense efforts, a validated, predictive biomarker for outcome to bevacizumab remains elusive. Given that bevacizumab not only plays major role in tumor vessels but also modulates tumor environment, suitable biomarkers for bevacizumab may be not limited to a single factor.

DNA methylation could represent gene networks regulation on the epigenetic level, which might better reflect the complex gene interactions [17]. DNA methylation is the robust characteristic of genes, and results in long-term stable programming of the genome [18, 19]. Thus, DNA methylation is the potential biomarkers for cancer patients, and the significant differences of DNA methylation patterns were observed between patients with better prognosis and inferior prognosis from bevacizumab.

It’s worth noting that related genes of DMRs on promoter regions were significantly enriched in neutrophil mediated immunity. Previous studies have observed the association of neutrophil numbers and areas of intense tumor vascularity in vivo and vitro models [20, 21]. Neutrophil is a kind of immune cell packed with the composition of chemokines, proteases and growth factors [22]. The potential mechanism by which neutrophil promote tumor angiogenesis is that these cytokines and factors, such as VEGF, chemokines, matrix metalloproteinase, would have impact on tumor vessels when released into the tumor microenvironment, which might directly drive angiogenesis or active nearby angiogenic-driving factors. Our results indicated that these genes were also significantly enriched in neutrophil degranulation pathway except for VEGFA-VEGFR2 Signaling Pathway. Thus, the neutrophil mediated VEGF-independent angiogenesis might be the potential mechanism of bevacizumab resistance in NSCLC patients.

This study firstly established a DNA methylation signature and investigated its prognosis prediction for bevacizumab in NSCLC patients. Using DNA from primary tumor tissues, we constructed a 10-gene methylation signature with a high predictive value for bevacizumab outcome in NSCLC patients, and patients with high score were associated with adverse clinical outcome. The host genes involved in the signature was found to be related to prognosis and immune micro-environment of cancer patients. *VTCN1* is known as an immune checkpoint of B7 superfamily of co-stimulatory molecules [23], and the expression of *VTCN1* on tumor associated macrophage was found to be related to the angiogenesis [24]. Increased *TTC21A* has been found to correlate with favorable prognosis and increased proportion of immune cells in patients with lung adenocarcinoma [25]. *PTH1R* was also found to induce *VEGF* expression supported HUVEC proliferation and migration [26].

Of interest, functional analysis indicated that host genes were enriched in neutrophil mediated immunity, and some of the host genes of DNA methylation signature were also found to be related to immune environment. The important role of immune environment in bevacizumab resistance has been studied a lot [27]. To better understand the implications of DNA methylation signatures, we then sought to explore the association between host genes and the abundance of immune cells from LUAD patients in TISIDB database. We found the significant abundance of ActCD4, Th1, ActCD8 cells, NKT and neutrophil cell be related to methylation of most of the host genes in the signature. Thus, we proposed that the potential biology basis of DNA methylation signature was associated with tumor immune environment.

Our present study has some limitations. Firstly, this was a retrospective study, and selection bias was inevitable. Secondly, another independent cohort of NSCLC patients receiving bevacizumab which is performed with DNA methylation assay will be required to validate out observation. The independently validation will be performed in our further studies. Thirdly, the biologic mechanism of DNA methylation signature remains to be further investigated.

## Conclusion

In summary, we report the significant difference in DNA methylation profiles between NSCLC patients receiving bevacizumab with inferior and better prognosis. And host genes of DMR were found to be enriched in neutrophil mediated immunity and neutrophil degranulation. We established a DNA methylation signature consisting of ten host genes as a predictive tool for selecting patients who stand to achieve clinical benefit from bevacizumab, which help to optimize treatment strategies for NSCLC patients.

## Method

### Patient selection and data collection

Twenty patients with advanced (IIIB/IV stage) non-squamous NSCLC receiving bevacizumab were eligible to enter the study from June 2015 to February 2020. The eligibility criteria were as follows: (1) pathological diagnosis of non-squamous NSCLC, (2) exposure to bevacizumab and chemotherapy treatment as first-line treatment, (3) accessible tumor samples before bevacizumab treatment, (4) available medical records. This study was performed in accordance with the principles of the 1975 Declaration of Helsinki and its later amendments or comparable ethical standards, and was approved by the Ethics Committee of Shandong Cancer Hospital.

The medical records of each patient were reviewed with respect to age, gender, EGFR status, smoking history, anatomical types, the presence of liver, brain, and bone metastasis, and laboratory complete blood count. Genomic DNA was purified from the FFPE tumor sample using GeneRead DNA FFPE Tissue Kit (Qiagen, 180,134).

The outcomes of survival analysis were PFS and OS. PFS was defined as the time from the start of bevacizumab to the progression event (according to Response Evaluation Criteria in Solid Tumors (RECIST) version 1.1) or death. OS was defined as the time from the start of bevacizumab to death. Patients with durable clinical benefit with bevacizumab (defined as no progression within the first 10 months of bevacizumab) were classified as better prognosis group (A group), and patients with progression event or death within the first 7 months of bevacizumab were assigned to inferior prognosis group (B group).

### Methylation data analysis

Genomic DNA was sonicated using bioruptor resulting in fragments of 200 bp (range: 100-400 bp). Adapter ligation was performed using NEBNext Ultra II DNA Library Prep Kit for Illumina (NEB, E7645). The product was then subjected to PCR in order to generate the whole MeDIP-seq Library, including immunoprecipitation with 5-Methylcytosine (5-mC) Monoclonal Antibody (Epigentek, A^− 1014^), amplified using Q5 High-Fidelity DNA Polymerase (NEB, M0491) and purified with AMPure XP beads (Beckman). Subsequently, the library was evaluated with Bioanalyzer 2100 (Agilent Technologies) and sequenced by Illumina Hiseq 4000.

Raw reads filtering from library was performed to filter out sequencing adapters, short reads (length < 35 bp) using Cutadapt v1.18 [28] and Trimmomatic v0.38 [29], and sequencing quality was assessed using FastQC software. Then the high-quality clean reads were aligned to a reference genome (hg38, GRCh38) using the Bowtie2 v2.3.4.1 [30]. After local realignment, peaks sites were identified from peak calling analysis using the MACS v2.1.2 [31] with 0.05 set as the q-value cutoff and annotated using ChIPseeker R package. Comparison of peaks sites between A group and B group were identified using DESeq2 method in DiffBind package, and peaks sites with FDR < 0.01 and |log2(fold change)| > 1 were selected as DMRs.

### Survival analysis and functional enrichment analysis between A and B group

Univariate cox analysis was performed to the determine the survival-related host genes of promoter-associated differentially methylated regions (DMRs). Functional enrichment analysis of survival-related host genes, including gene ontology (GO) analysis and pathway analysis from KEGG Pathway, Reactome Gene Sets and WikiPathways, was performed using the Metascape web-based tool [32].

### Construction and validation of genome-wide DNA methylation signature

The LASSO-Cox analyses were utilized to identify independent DNA methylation-driven genes that were significantly associated with prognosis of bevacizumab. Genome-wide DNA methylation signature was constructed based on the risk coefficient and DNA methylation-driven genes. Univariate and multivariate cox analyses were performed to validate the prognostic role of DNA-methylation signature and identify the independent variables of survival. The relationship between DNA methylation signature and patient characteristics estimated with χ^2^ test. To reveal the related immune infiltration of DNA methylation signature and immune infiltration, patients with lung adenocarcinoma (LUAD) in TISIDB database was used to infer the relations between abundance of tumor-infiltrating immune cells and DNA methylation-driven genes [33].

### Statistical analyses

All statistical analyses were implemented using R version 3.4.4 and SPSS version 24.0. The “glmnet” package was used to perform the LASSO-Cox model. And Kaplan-Meier survival and Cox proportional hazards regression, with the differences calculated with the log-rank test were performed using the “survival” package. All statistical tests were two-sided, and *P* values less than 0.05 were considered statistically significant.

## Supplementary Information

**Additional file 1.**

## Data Availability

The datasets generated during the current study are available in the National Genomics Data Center with the accession number of HRA002505.
