# Supplementary material for: Genome-wide DNA methylation signature predict clinical benefit of bevacizumab in non-small cell lung cancer
Source: BMC Cancer. 2022 Jul 29;22:828. doi: 10.1186/s12885-022-09918-1 (PMC9338664; doi:10.1186/s12885-022-09918-1)
Supplement: Supplementary file 1 — Additional file 1. [file 12885_2022_9918_MOESM1_ESM.docx]

**
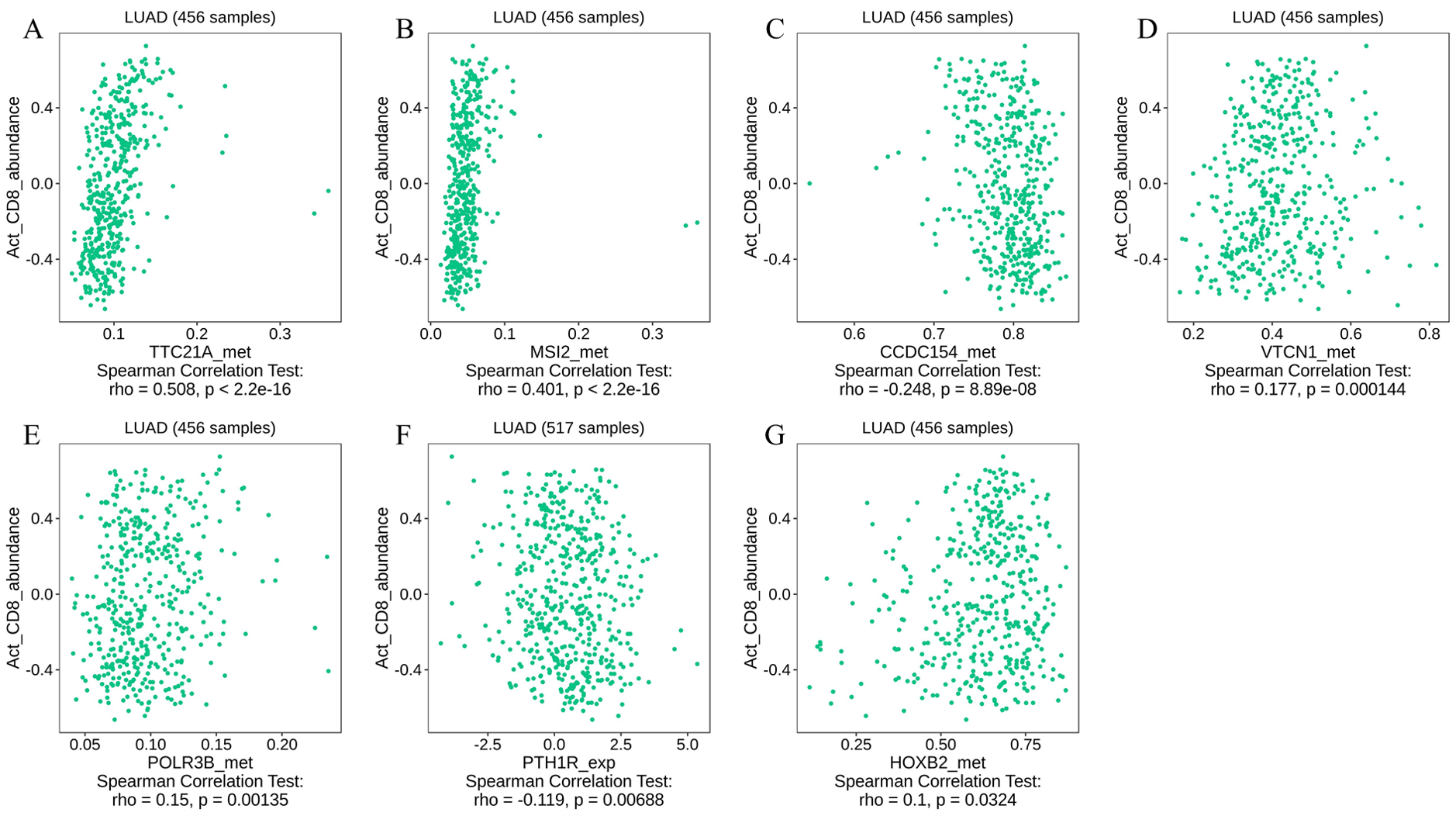
Supplementary Figure 1. The association between abundance of ActCD8 cell and host genes in DNA methylation signature.** A. *TTC21A*. B. *MSI2*. C. *CCDC154*. D. *VTCN1*. E. *POLR3B*. F.*PTH1R*. F. *HOXB2*.


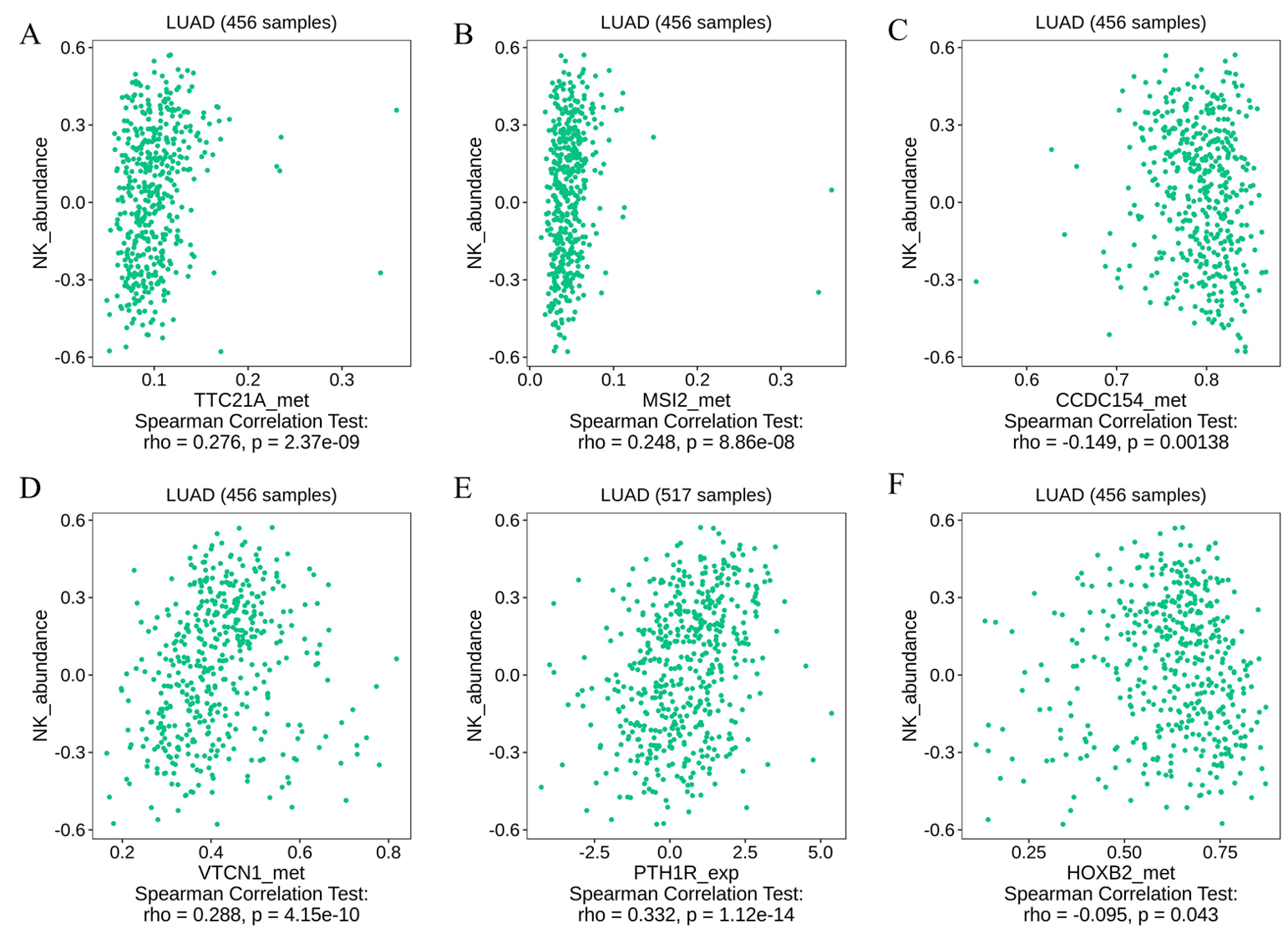


**Supplementary Figure 2. The association between abundance of NKT cell and host genes in DNA methylation signature.** A. *TTC21A*. B. *MSI2*. C. *CCDC154*. D. *VTCN1*. E. *PTH1R*. F. *HOXB2*.
